# Food Availability, Motivational-Related Factors, and Food Consumption: A Path Model Study with Children

**DOI:** 10.3390/ijerph182412897

**Published:** 2021-12-07

**Authors:** Beatriz Pereira, Pedro Rosário, José Carlos Núñez, Daniela Rosendo, Cristina Roces, Paula Magalhães

**Affiliations:** 1 Department of Applied Psychology, University of Minho, 4710-052 Braga, Portugal; beatriznpereira94@gmail.com (B.P.); prosario@psi.uminho.pt (P.R.); danielapatriciamr@gmail.com (D.R.); 2 Department of Psychology, University of Oviedo, 33003 Oviedo, Spain; jcarlosn@uniovi.es (J.C.N.); croces@uniovi.es (C.R.)

**Keywords:** food consumption, healthy eating, motivational-related factors, food availability, self-regulation, self-efficacy, children, path model

## Abstract

The promotion of children’s healthy eating is a key public health priority. However, children’s food consumption is a complex phenomenon with several contributing factors, and there is a call to continue developing comprehensive models with several variables acting simultaneously. The present study aimed to examine the role different motivational-related variables (e.g., self-regulation, self-efficacy) may play in children’s consumption of healthy and unhealthy foods. To address this goal, data were collected in a sample of 242 fifth and sixth graders with access to both healthy and unhealthy foods at home. A path model was conducted to analyze networks of relationships between motivational-related variables and children’s healthy and unhealthy eating. The gender variable was included as a covariate to control its effect. The data showed that self-regulation for healthy eating mediates the relationship between the predictor variables (i.e., knowledge, attitude, and self-efficacy) and the type of food consumption (healthy and unhealthy). Current data contribute to understanding the complexity behind food consumption by providing a comprehensive model with motivational-related factors associated with both healthy and unhealthy eating. The present findings are likely to help inform the development of early preventive interventions focused on the promotion of healthy eating.

## 1. Introduction

Promoting children’s healthy eating is a key public health priority to prevent chronic diseases (e.g., obesity, diabetes) and maintain health, well-being, and school performance [1,2,3]. To help achieve this public health goal, international organizations have been establishing eating guidelines for children, advocating the intake of five pieces of fruits and vegetables (F/V) per day and the limitation of fat and sugar consumption [1,2]. However, there is extensive evidence that children are not meeting the recommendations and are instead consuming large amounts of energy-dense foods, fat, and sugar, and low amounts of F/V [1,3,4,5]. Particularly, it seems that boys have unhealthier diets when compared with girls [5,6,7]. 

To find additional solutions for this unhealthy eating scenario, predictors of children’s food consumption have been a major research target over the past few decades. Many variables have been studied, including variables that are external and not amenable to be changed by the individual (e.g., food economy–high prices of F/V) and child-related variables that are amenable to be changed by the individual (e.g., nutritional knowledge) [8,9,10]. Although it is well established that external variables greatly affect children’s food consumption, exclusively relying on them to predict food consumption does not account for the agential role of the child. In fact, by considering only the influence of external variables on individuals’ eating behaviors, the individuals’ efforts to develop adaptive competences and skills for adopting and maintaining healthy eating behaviors are not considered (e.g., choosing a healthy snack by themselves alongside the external constraints) [11,12]. 

Drawing on this premise, researchers are increasingly giving attention to the role of child-related factors, especially motivational-related ones, on children’s food consumption [13,14]. For example, regarding child-related factors, studies in the literature have been extensively investigating the relationship between nutritional knowledge and children’s food consumption. While some studies have shown no relationship between nutritional knowledge and children’s dietary practices, most research has emphasized that nutritional knowledge seems to be necessary, or at least important, for making healthy food choices [9,10,15]. As a matter of fact, knowledgeable individuals seem to be 25 times more likely to consume adequate amounts of F/V daily than unknowledgeable ones [16]. Concerning the specific case of motivational-related factors, the role of self-efficacy on children’s food consumption is also well established. In fact, the literature suggests that high self-efficacy regarding eating behaviors is associated with a high consumption of F/V and low consumption of fat, whereas low self-efficacy is associated with unhealthy eating patterns [17,18]. Furthermore, researchers have also been studying the role that children’s attitudes and perceptions toward healthy eating play on food consumption. Actually, healthy eating seems more likely to be adopted by children who think and reflect on the long-term benefits of healthy foods [19]. Moreover, research shows that attitudes about healthy eating (e.g., thinking that broccoli is good for health) have a stronger influence on eating behavior than declarative knowledge about food [20].

Although the research acknowledges the role played by these child-related factors on children’s food consumption, little is known about the factors and mechanisms underlying these relationships. A systematic review suggested that, among other factors, using self-regulation (SR) strategies may be key for individuals to adopt healthy eating behaviors [14]. For example, adolescents who frequently use SR strategies (e.g., putting chips out of reach when watching television, setting goals about how many candies a day one can have) consume fewer unhealthy foods [21]. In addition, improvements in SR are associated with an increase in F/V consumption [22]. This may be explained by the use of SR allowing individuals to proactively take control of the personal, behavioral, and environmental influences that impact human behavior, including eating [23]. Thus, it is possible that self-regulation may help in understanding the mechanisms likely explaining the relationship between knowledge, self-efficacy, and attitudes toward healthy eating and children’s food consumption. In fact, a recent study showed that knowledge about healthy eating only influenced children’s weight when the children with knowledge on healthy eating also used SR strategies. [13]. Furthermore, research shows that children and adolescents with a high self-efficacy were prone to using a diverse spectrum of SR strategies [24]. Moreover, recent research [25] conducted structural equation modeling to examine the role of a set of variables (i.e., nutrition-related self-efficacy, intentions to eat F/V, behavioral strategies, outcome expectations, outcome expectancies, situation, and social support) on adolescents’ reported consumption of F/V. Findings indicated that adolescents’ intentions to eat F/V mediated the relationship between self-efficacy, social support, and outcome expectancies and F/V consumption behavior. This could be due to the fact that children need to have confidence and mastery of their ability, i.e., self-efficacy, to implement SR strategies to control the personal and external influences on behavior [26,27]. Lastly, it seems that attitudes also affect the use of SR strategies. For example, when individuals understand the utility of using SR strategies to achieve their self-set goals, they are more likely to use them and achieve success [28]. 

Taking this information all together and grounding on previous research of child-related factors (especially motivational-related ones) that may contribute to children’s food consumption, the present study aimed to improve our understanding of the following aspects: (i) the mediator role played by SR toward healthy eating in the relationship between the predictor variables (i.e., declarative knowledge, self-efficacy, and attitudes toward healthy eating) and the (un)healthy food consumption of elementary-school children; and (ii) the role played by gender in the relationship between SR and children’s food consumption. Children were chosen as participants in this study because the formation of habits and behaviors occurs during childhood [29]. Moreover, as habits developed during childhood are likely to persist through adulthood, it is important to intervene before challenges arise [30]. 

**Figure 1:**
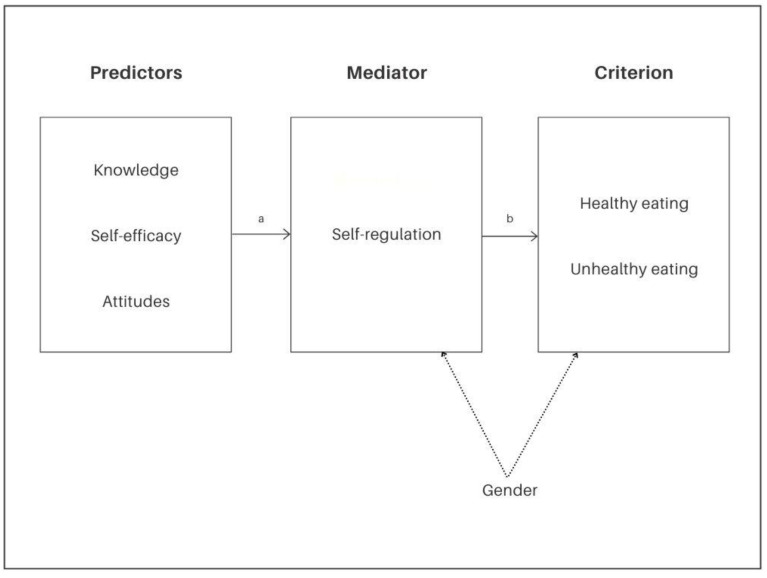
Explanatory model of the mediational role of the self-regulation processes toward healthy eating on the relationship between the predictor factors (i.e., declarative knowledge about healthy eating, self-efficacy processes toward heathy eating, and attitudes and perceptions on healthy eating) and the food consumption criterion variables (healthy and unhealthy eating). Note. Coefficients a and b are expected to be statistically significant; gender was treated as a covariate to statistically control its effect.

The present paper's novelty arises from its investigation of the extent to which the collected data support a complex net of relations among multiple children-related factors, especially motivational-related ones, expected to help explain children’s food consumption. Although recent studies in the literature have started investigating motivational-related factors as important drivers of children’s (un)healthy eating, little investigation has been performed on how factors interact with each other (i.e., research tends to evaluate one or two factors in isolation) [17]. Examining only separate elements risks not understanding the real contribution of each factor to the phenomenon [31]. Thus, analyzing all the variables' relationships within the same model is expected to contribute to a more comprehensive understanding of the role played by these variables on the control an individual may possess in the complex phenomenon of food consumption [13,14]. Learning the relationships between factors using a path model is expected to provide valuable information on the process and help design multidimensional preventive interventions aiming to promote healthy eating. Toward this goal, a mediation model (Figure 1) was devised with the following hypothesis:

The current model hypothesized that the predictor variables (i.e., declarative knowledge about healthy eating, self-efficacy processes toward heathy eating, and attitudes and perceptions on healthy eating) are indirectly related to children’s food consumption (i.e., healthy and unhealthy eating) through their relationship with self-regulation processes toward healthy eating. The latter association is expected to be: (i) positive for healthy eating: the higher the self-efficacy, attitudes, and knowledge scores, the higher the use of SR strategies, and the higher the likelihood of children’s healthy food consumption; and (ii) negative for unhealthy eating: the lower the self-efficacy, attitudes, and knowledge scores, the lower the use of SR strategies, and the higher the likelihood of children’s unhealthy food consumption.

## 2. Materials and Methods

### 2.1. Study Context

The study took place in five public schools in the North of Portugal from different settings/contexts (e.g., rural, urban). In addition, the children attending the schools enrolled are likely to come from a wide range of socioeconomic and cultural backgrounds. Importantly, children from varied socioeconomic and cultural backgrounds are expected to have distinct resources and opportunities to make food choices. Considering the aim of the present study, i.e., to contribute to the understanding of how child-related factors influence children’s food consumption, only children with food availability at home were recruited. The reason for this inclusion criteria was that children without food availability are limited in their ability to control their food choices (e.g., if they do not have fruit at home, the likelihood of choosing to eat fruit at lunch decreases). In fact, while analyzing individuals’ choices, researchers cannot disregard the role that food availability at home might play [14,32,33]. Research shows that having a limited variety of foods available at home may inhibit a child’s ability to choose and, consequently, affect the quality of their food consumption [32,33]. Specifically, the likelihood of selecting a healthy or unhealthy snack is lower when the availability of these types of food is scarce [34]. Therefore, following the literature’s warnings, the present study comprised children from diverse socioeconomic and cultural backgrounds with access to both healthy (i.e., F/V) and unhealthy (i.e., candies and refrigerants, and fats and fast-food) foods at home. 

### 2.2. Participants and Procedure

The present study is part of a research project that has been approved by the University of Minho Ethics Committee for Research in Social and Human Sciences (CEICSH) (CEICSH 032/2019). CEICSH confirmed that the project complies with the requirements for good practice in human research, being in accordance with national and international standards governing research in social and human sciences, including the Declaration of Helsinki. Additionally, consent to conduct the study was obtained from the Portuguese Ministry of Education. 

**Figure 2:**
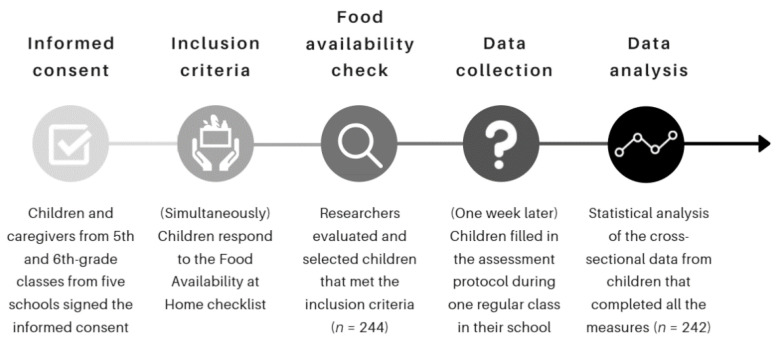
Timeline of the data collection procedure.

Prior to data collection, children and caregivers were informed by the research assistants about the aims of the study and were assured of the confidentiality of the data. Children and caregivers' written informed consent agreements were requested in 5th and 6th-grade classes from the five schools participating in the study. During this phase, participants who met the food availability inclusion criteria were selected (i.e., children who declared having both healthy and unhealthy foods at home; see the instruments and measures section for more details on food availability assessment). Thus, 244 elementary school students met the inclusion criteria (i.e., consent to participate and have food availability at home) and took part in the data collection. Data collection took place in regular classes between January and February 2018. Students were invited by a research assistant to fill in the instruments and were asked to complete the task by themselves. Children took approximately 40 min to complete the questionnaires; each child fulfilled the instruments at their own pace and was supported by the research assistant with any item found to be unclear. From the initial pool of participants, 2 (0.82%) had to be excluded due to the lack of completion of all the measures collected (e.g., did not answer to the SR scale). The above data collection procedure is presented in Figure 2. Thus, the final sample was composed of 242 children aged between 9 and 12 (M = 11; SD = 0.797). Of these participants, 107 (44.2%) were female. 

### 2.3. Instruments and Measures

#### 2.3.1. Personal Data

Participants were asked about their gender, age, and school grade.

#### 2.3.2. Food Availability at Home

To assess food availability at home, a checklist comprised of eight food groups was used [5]. The checklist consisted of eight images (i.e., cereals, derivatives, and tubers; vegetables; fruits; legumes; dairy products; meat, fish, and eggs; candies and soda; fats and fast food), and children were asked to circle the groups of foods they had available at home. For the purposes of the current study, children only met the food availability inclusion criteria when they reported access to both healthy (i.e., vegetables and fruits) and unhealthy (i.e., candies and refrigerants, and fats and fast-food) food groups.

#### 2.3.3. Self-Regulation Processes toward Healthy Eating Questionnaire

To evaluate the SR processes toward healthy eating, the Self-Regulation Processes towards Healthy Eating Questionnaire [13] was used. The scale consisted of nine statements regarding the participants’ use of SR strategies toward healthy eating (e.g., I plan my meals. I think about what I am going to eat and what it takes to prepare my meal; (for example, after waking up, I think about what I will eat for breakfast and what I need to prepare it)). Responses to the individual items were scored from 1 (never) to 5 (always) on a Likert-like scale and summed to create a composite score that ranged from 9 to 45, with higher scores implying more SR. The reliability of the scale is good (α = 0.788; ω = 0.792).

#### 2.3.4. Self-Efficacy Processes toward Healthy Eating Questionnaire

To evaluate self-efficacy processes toward healthy eating, an adapted version of the Self-Efficacy for Health Scale [35] was used. This adapted scale consisted of twenty statements regarding situations where the participants were asked to evaluate their confidence about being able to choose a healthy option (e.g., Eat fruit at breakfast). Responses to the individual items were scored from 1 (cannot do at all) to 4 (highly certain can do) on a Likert-like scale and summed to create a composite score that ranged from 20 to 80, with higher scores implying more self-efficacy. The reliability of the scale is good (α = 0.832; ω = 0.835).

#### 2.3.5. Students' Attitudes and Perceptions on Healthy Eating Questionnaire

To evaluate the participants’ attitudes and perceptions about healthy eating, an adapted version of the Students' Attitudes and Perceptions on the Health Instrument [35] was used. The present instrument consisted of 17 statements about students’ attitudes and perceptions about the importance of healthy eating (e.g., Eating fruit and vegetables will help me to grow up; I think fruit is tasty). Responses to the individual items were scored as true or false, and the answers marked to be true were summed to create a composite score that ranged from 0 to 17, with higher scores implying more positive attitudes and perceptions toward healthy eating. The reliability of the scale is moderate (KR-20 = 0.626).

#### 2.3.6. Declarative Knowledge about Healthy Eating Questionnaire

To evaluate the participants’ declarative knowledge about healthy eating, an adapted version of the Declarative Knowledge about Healthy Eating Questionnaire [36], initially developed for assessing declarative knowledge about health in general (e.g., healthy eating, oral health), was used. The final scale consisted of 10 statements about healthy eating (e.g., It is necessary to eat fruits and vegetables, but not every day). The answers to the individual items were scored as being true or false; the correct answers were summed to create a composite score that ranged from 0 to 10, with higher scores meaning more declarative knowledge about healthy eating. The reliability of the scale is moderate (KR-20 = 0.547).

#### 2.3.7. Previous Day Food Intake Questionnaire (PDFIQ)

The Previous Day Food Intake Questionnaire [37] is a visual instrument used to assess the foods children had eaten during different meals throughout the previous day (i.e., breakfast, mid-morning snack, lunch, afternoon snack, dinner, and before-bed snack). The scale consisted of six images, one for each meal, illustrated with 21 individual foods and some food groups, specifically: dry beans, rice, milk, coffee with milk, chocolate milk, cheese, yogurt, beef or poultry, pasta, bread or crackers, French fries, pizza or hamburger, leafy vegetables, starchy vegetables, vegetable soup, fruits, sweets, chips, fish/sea foods, soft drinks, and fruit juices [37]. For each image of a meal, children were asked to circle the foods that they had eaten during the previous day for the corresponding meal. The instrument’s reliability in evaluating the foods consumed was 70.2% and was 96.2% for foods not consumed. For the present study, responses for each image were scored based on the frequency at which these foods were consumed throughout the day, i.e., in how many meals had the children consumed fruit and vegetables, and in how many meals had the children consumed fast-food, soda, and candies. The reliability of the scale is moderate for healthy (KR-20 = 0.673) and unhealthy food intake (KR-20 = 0.619).

### 2.4. Data Analysis

The data were analyzed in three stages. First, we analyzed the statistical properties of the variables included in the path model (means, typical deviations, asymmetry, kurtosis), as well as the correlation matrix and the missing values (the percentage of missing values in the final sample was low, about 0.80%). Secondly, the healthy and unhealthy eating path models were fit with the AMOS 22 program in SPSS [38], using the robust maximum likelihood (ML). In this model, the gender variable was included as a covariate in order to statistically control its effect on the explanation of the variability of the dependent variables (i.e., SR, healthy and unhealthy eating). In fact, the literature shows that girls use more SR strategies than boys and that boys eat unhealthier than girls, regarding the amount of fruit and vegetable intake [5,7,39]. Model fit was evaluated using χ^2^, χ^2^/df, the adjusted goodness-of-fit index (AGFI), the comparative fit index (CFI), the root-mean-square error of approximation (RMSEA), and the standardized root-mean-square residual (SRMR). There is evidence of a good fit when χ^2^ has p > 0.05, χ^2^/df < 5, AGFI ≥ 0.90, CFI ≥ 0.95, and RMSEA and SRMR ≤ 0.05. Finally, a multiple-group analysis was conducted to verify whether the fit model was invariant for the sex variable. The effect size of the regression coefficients was calculated using Cohen’s [40] d statistic.

## 3. Results

### 3.1. Descriptive Statistics

Descriptive data are presented in Table 1. Most of the correlations are statistically significant. It is particularly striking that there is no relationship between healthy and unhealthy eating. The predictor variables (i.e., knowledge, attitudes, self-efficacy, and self-regulation) show a normal distribution. Self-regulation, self-efficacy, knowledge, and attitudes are positively associated with healthy eating and negatively with unhealthy eating. The correlations indicate that healthy eating tends to be associated with more positive attitudes, more self-efficacy, and the use of SR strategies for healthy eating. Conversely, unhealthy consumption is associated with more negative attitudes, less knowledge, less self-efficacy, and fewer SR strategies. Finally, despite girls reporting more healthy eating consumption than boys (i.e., positive correlation between gender and healthy eating. Note that boys are coded with zero and girls with one), no relationship between gender and unhealthy consumption was found.

### 3.2. Explanatory Model of Food Consumption (Healthy and Unhealthy Eating Models)

While the healthy eating model presents an excellent fit (χ^2^(3) = 2.128; *p* = 0.546; χ^2^/*df* = 0.709; AGFI = 0.980; CFI = 0.999; RMSEA = 0.001; SRMR = 0.018), the unhealthy eating model fit is just acceptable (χ^2^(3) = 11.835; *p* = 0.008; χ^2^/*df* = 3.945; AGFI = 0.890; CFI = 0.951; RMSEA = 0.111; SRMR = 0.044). Table 2 presents the standardized regression coefficients for healthy and unhealthy eating models (direct and indirect effects). According to Table 2, in both the healthy and unhealthy eating path models, there is a significant positive relationship between attitudes and self-regulation, self-efficacy and self-regulation, and gender and self-regulation. The relationship between knowledge and self-regulation and between gender and (un)health was not significant. Moreover, self-regulation positively influenced children’s healthy eating and negatively influenced children’s unhealthy eating. For indirect effects, the data showed that attitudes and self-efficacy positively explained healthy eating through self-regulation. Regarding unhealthy eating, only self-efficacy negatively explained children’s food consumption through self-regulation. 

**Figure 3:**
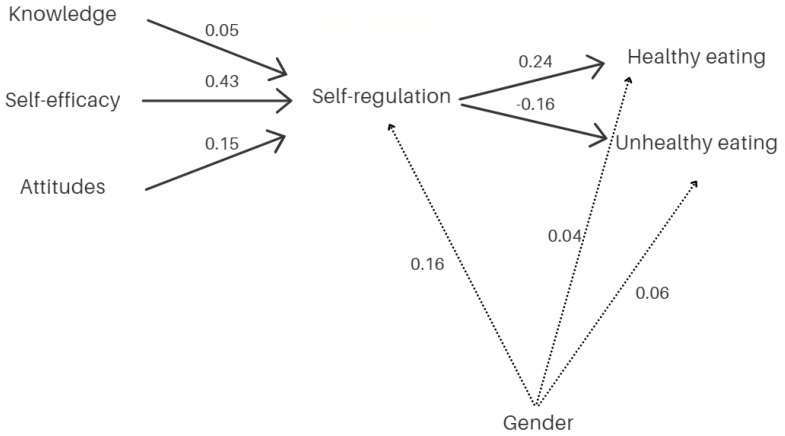
Principal results of the food consumption model.

Figure 3 presents the principal results. The relevance of controlling the gender variable has been validated. Specifically, gender did not directly affect the type of food consumption (neither healthy nor unhealthy), but rather indirectly through its effect on SR (see Figure 3).

Our main hypothesis was that SR for eating behaviors plays a mediating role between self-efficacy, attitude, and knowledge on healthy and unhealthy eating. The data obtained confirm this hypothesis only partially. SR for healthy eating is positively associated with healthy eating (*b* = 0.244, *p* < 0.001) and negatively with unhealthy eating (*b* = −0.164, *p* < 0.01). Moreover, SR for eating behaviors is positively predicted by children’s self-efficacy (*b* = 0.432, *p* < 0.001) and their positive attitude (*b* = 0.149, *p* < 0.01), but not by their level of knowledge toward healthy eating (*b* = 0.049, *p* = 0.375). However, considering the indirect effects (see Table 2), the SR mediation hypothesis is confirmed only for some relationships. Specifically, considering the healthy food model, the SR mediation was statistically significant for the effects of attitude (*b* = 0.036, *p* < 0.05) and self-efficacy (*b* = 0.106, *p* < 0.001), but not for knowledge (*b* = 0.012, *p* > 0.05). Regarding the unhealthy food model, only the SR mediation for self-efficacy was statistically significant (*b* = 0.071, *p* < 0.05). It should also be noted that a total mediation effect was found in cases where the mediation effect was significant; that is, the effect of the independent variables on eating behavior—healthy or unhealthy—was entirely through SR (note that there are no statistically significant direct effects of knowledge, self-efficacy, or attitude on eating behavior). However, the effect sizes of such mediation effects are small (note that only the mediation of the effect of self-efficacy on healthy eating is close to medium: *d* = 0.452).

Finally, 34.3% of the variance of SR for eating behaviors is explained by the predictor variables, but healthy and unhealthy eating are barely explained (7.3% and 2.4%, respectively).

## 4. Discussion

The present study analyzed the reported food consumption behavior of children from the 5th and 6th grades with food availability at home. A path model was tested to investigate how the self-regulation of healthy eating mediates the effect of child-related variables (i.e., knowledge, self-efficacy, and attitudes) on food consumption. Finally, gender was controlled due to its potential effect on SR and the intake of healthy and/or unhealthy food. 

Overall, the hypothesized mediation model was partially confirmed, i.e., some motivational-related factors are related to children’s (un)healthy food consumption through the strategies that children use to self-regulate their eating.

The direct effects of the present model showed that attitudes and self-efficacy toward healthy eating were positively associated with self-regulation toward healthy eating, i.e., children who think positively about healthy eating and feel capable of making healthy food choices are more likely to self-regulate their eating behaviors. Woods et al. [25] also found that self-efficacy can lead to an increase in behavioral strategies (e.g., tracking food consumption, planning meals) aimed toward fruit and vegetable consumption. Moreover, in the current model, self-efficacy showed a positive effect on SR stronger than that found for attitudes about healthy eating. This finding emphasizes the prominent role children’s beliefs about their capability to perform a task play in carrying out that task [26,27,41,42]. Thus, it seems necessary to promote children's sense of confidence in their ability to use SR strategies for healthy eating to promote their adherence to healthy eating. Interestingly, there is no association between declarative knowledge about healthy eating and SR for healthy eating. 

Still, regarding the direct effects of the model, SR toward healthy eating was positively associated with children’s food consumption, i.e., high SR strategies are associated with healthier food choices, and poor SR strategies are associated with unhealthier food choices. These findings are consistent with previous research showing that SR behaviors may play a relevant role in developing a healthy diet [13,14]. This stresses the importance of the child’s agent role in managing and activating personal resources to make healthy food choices (e.g., plan a healthy school lunch box). 

A detailed analysis of the indirect effects (mediational hypothesis) indicates that the effects of self-efficacy and attitudes toward healthy eating are fully mediated by SR. The former exhibited a medium effect size while the latter showed a small effect size. In fact, the more positive the attitude toward and the self-efficacy for healthy eating, the higher the SR and the healthier the eating choices made. Conversely, the effect of self-efficacy for healthy eating on the consumption of unhealthy food is negative. Importantly, the stronger the self-efficacy, the higher the protective effect on the consumption of unhealthy food. This effect is similar regarding attitudes about healthy eating, but the impact is marginally significant. These indirect effects emphasize the idea that food consumption is a complex phenomenon with a network of relationships between several contributing factors [14,25]. Interestingly, SR for healthy eating does not mediate the effect of knowledge on healthy food consumption. This relevant finding supports prior evidence suggesting that solely conveying knowledge about healthy eating is insufficient to influence healthy food choices being made [13].

Lastly, gender did not directly impact food consumption but instead showed an indirect impact through SR. Girls in the present sample reported more self-regulation behaviors than boys, which in turn may lead to girls consuming more healthy foods and less unhealthy ones than boys. These data help in understanding the mechanisms underlying the gender differences regarding food consumption found in previous studies [5,7]. 

The current study’s promising results sustain the idea that multidimensional interventions and actions are needed to promote eating behavior changes, rather than interventions focused on a single factor in isolation [14]. Grounded on present data, we believe that motivational-related factors are key in the promotion of healthy eating, but also that their impact on children’s food consumption is lower than expected. Therefore, the design of future research and interventions might consider: (a) promoting SR toward healthy eating (e.g., hands-on activities about meal planning) [23,36,43]; (b) boosting feelings of self-efficacy toward healthy eating (e.g., group interventions where children discuss their experiences in making (un)healthy choices with their peers, reinforcing the feasibility of success) [44]; (c) promoting positive attitudes toward healthy eating (e.g., illustrating that increasing fruit and vegetable consumption is important not only to maintain a healthy weight, but also to protect against chronic diseases) [45]; (d) improving the explained variance of food consumption by considering the inclusion of social-related variables in the path model (e.g., peer pressure on food consumption [46]).

Additionally, it is important to use current outcomes to inform the activities of groups comprised of children, parents, teachers, and the health community in the future and advocate for the importance of launching policies promoting children’s agency toward healthy eating. Advocacy groups for healthy eating could consider working with governmental agencies and food companies to help set environmental and structural changes likely to facilitate children’s healthy choices (e.g., training for parents on healthy meal preparation, delivering media marketing messages encouraging healthy food choices, or lowering prices for healthy foods). Although the present research advances the understanding of how motivational-related factors may contribute to children’s food consumption, the findings should be taken cautiously. For example, no association between declarative knowledge about healthy eating and SR for healthy eating was found. This important finding is expected to provide further insight to school administrators and healthcare regulatory agencies on the limited or small-to-medium-sized effects of healthy food campaigns [14,47,48], as well as suggestions for future interventions (e.g., include self-efficacy and attitudes toward healthy eating). Moreover, despite the effect of SR on food consumption being statistically significant, the amount of explained variance is small. The following reflections could shed light on current findings. Participants are elementary school children declaring access to both healthy and unhealthy foods at home. This is a strength of the present study, as it ensures the exclusion of children that would not have the possibility to make choices regarding their eating habits, due to lack of accessibility; yet, due to their early age, the participants may still lack autonomy in their food choices. For example, some parents set limits, rules, or restrictions regarding food consumption [49]; moreover, children could eat some of their meals at school and may purchase (un)healthy items in the cafeteria [50]. In sum, current data show that children’s SR may play a limited role in their food choices, and there is a need to include other variables in the model to increment the explained variance of (un)healthy food consumption (e.g., external factors such as parental styles and school cafeteria vending options) [51,52]. At this age, although motivational-related factors seem to play an important role in food consumption, external factors could also play a key role in food choices, and therefore need to be added to the model. 

Moreover, a few limitations must be acknowledged. First, the self-reported nature of the measures used in the research may have led to socially desirable or inaccurate responses. Despite the reliability and validity of the measures, future research could consider combining self-reported with alternative measures, such as objective observational ones (e.g., children sending photos of the meals). Second, the data are cross-sectional. Future research could consider a longitudinal design to examine whether changes in any of the motivational-related factors predict actual behavior changes. Finally, although it is not possible to guarantee the generalization of the present findings, current data were collected in five public schools from different settings/contexts (e.g., rural, urban). This variety of settings ensured a wide range of socioeconomic and cultural backgrounds.

## 5. Conclusions

Overall, the results of the present study contribute to the existing literature, shedding light on the role of motivational-related factors on children’s food consumption. To further understand the etiology of (un)healthy food consumption from a motivational perspective, we evaluated the mediating role of self-regulation on the relationship between knowledge, self-efficacy, and attitudes toward healthy eating and children’s reported food consumption. The present results showed that self-efficacy and attitudes indirectly explain both healthy and unhealthy eating through self-regulation strategies. These findings are expected to help inform the design of future multidimensional interventions promoting healthy eating behaviors. For example, educators could consider instigating feelings of self-efficacy while encouraging the use of self-regulation strategies toward food choices. The study of a complex net of relationships among multiple motivational-related factors within the same model is an advantage of the present research. All considered, it is important to continue examining the complex relationships between different modifiable variables and their relationships with external factors (i.e., proximal and distal factors such as family meal climate and proximity to food outlets, respectively) to understand how to better-design preventive interventions likely to promote children’s healthy eating and, consequently, decrease childhood overweightness and obesity-related problems. Future research could consider the study of other motivational-related variables that may significantly influence children’s food consumption, for example, how parental styles and school vending options may impact children’s autonomy to choose and control their eating behaviors. Moreover, to address some limitations of the present study, future research could consider using observational measures and longitudinal designs.

## Figures and Tables

**Table 1:** Pearson correlations, mean, standard deviation, skewness, and kurtosis of observed measures (*N* = 242).

	Gender	Self-Regulation	Self-Efficacy	Knowledge	Attitude	Healthy Eating	Unhealthy Eating
Gender	–						
Self-Regulation	0.310 **	–					
Self-Efficacy	0.271 **	0.539 **	–				
Knowledge	0.226 **	0.223 **	0.253 **	–			
Attitudes	0.153 *	0.332 **	0.345 **	0.192 **	–		
Healthy Eating	0.138 *	0.264 **	0.199 **	0.091	0.163 *	–	
Unhealthy Eating	−0.008	−0.150 *	−0.164 *	−0.187 **	−0.183 **	0.028	–
M	0.44	32.39	66.47	8.31	12.29	2.19	1.96
SD	0.50	5.99	8.02	1.64	2.42	1.62	1.62
Skew	0.23	−0.83	−1.45	−0.98	−0.54	0.47	0.65
Kurt	−1.96	1.04	4.26	0.36	−0.25	−0.46	−0.46

Note. Sex (0 = boys; 1 = girls), Self-Regulation (9 = min.; 45 = max.), Self-Efficacy (20 = min.; 80 = max.), Knowledge (0 = min.; 10 = max.), Attitudes (0 = min.; 17 = max.), Healthy and Unhealthy eating (0 = min.; 6 = max.). * *p* < 0.05; ** *p* < 0.01.

**Table 2:** Standardized regression coefficients of the explanatory model of food consumption (*N* = 242).

	*SRW*	*S.E.*	*T*-Value	*p*-Value	*Cohen’ d*
Direct Effects					
Healthy Eating Path Model					
Knowledge → Self-Regulation	0.049	0.201	0.886	0.375	---
Attitudes → Self-Regulation	0.149	0.139	2.655	0.008	0.346
Self-Efficacy → Self-Regulation	0.432	0.043	7.447	<0.001	1.091
Self-Regulation → Healthy Eating	0.244	0.018	3.746	<0.001	0.496
Gender → Self-Regulation	0.160	0.664	2.897	0.004	0.379
Gender → Healthy Eating	0.062	0.212	0.945	0.344	---
Knowledge ↔ Attitudes	0.192	0.260	2.933	0.003	0.384
Knowledge ↔ Self-Efficacy	0.253	0.872	3.811	<0.001	0.505
Attitudes ↔ Self-Efficacy	0.345	1.318	5.066	<0.001	0.689
Unhealthy Eating Path Model					
Knowledge → Self-Regulation	0.049	0.201	0.886	0.375	---
Attitudes → Self-Regulation	0.149	0.139	2.655	0.008	0.346
Self-Efficacy → Self-Regulation	0.432	0.043	7.447	<0.001	1.091
Self-Regulation → Unhealthy Eating	−0.164	0.018	−2.443	0.015	0.318
Gender → Self-Regulation	0.160	0.664	2.897	0.004	0.379
Gender → Unhealthy Eating	0.043	0.217	0.637	0.524	---
Knowledge ↔ Attitudes	0.192	0.260	2.933	0.003	0.384
Knowledge ↔ Self-Efficacy	0.253	0.872	3.811	<0.001	0.505
Attitudes ↔ Self-Efficacy	0.345	1.318	5.066	<0.001	0.689
Indirect Effects					
Healthy Eating Path Model					
Knowledge → SR → Healthy Eating	0.012	0.014	0.865	0.387	---
Attitudes → SR → Healthy Eating	0.036	0.017	2.195	0.028	0.285
Self-Efficacy → SR → Healthy Eating	0.106	0.031	3.431	0.001	0.452
Unhealthy Path Model					
Knowledge → SR → Unhealthy Eating	−0.008	0.010	−0.836	0.403	---
Attitudes → SR → Unhealthy Eating	−0.024	0.013	−1.813	0.070	---
Self-Efficacy → SR → Unhealthy Eating	−0.071	0.030	−2.352	0.019	0.306

Note. *SRW* (Standardized Regression Weights); *S.E.* (Standard Error); → (Direction of relationship); ↔ (Direction of relationship); SR (Self-Regulation).

## Data Availability

Data are available from the first author upon reasonable request.
